# Therapeutic Implications of Targeting YY1 in Glioblastoma

**DOI:** 10.3390/cancers16112074

**Published:** 2024-05-30

**Authors:** Inesa Navasardyan, Apostolos Zaravinos, Benjamin Bonavida

**Affiliations:** 1 College of Osteopathic Medicine of the Pacific, Western University of Health Sciences, Pomona, CA 91766, USA; inesa.navasardyan@western.edu; 2 Department of Microbiology, Immunology & Molecular Genetics, University of California at Los Angeles, Los Angeles, CA 90095, USA; 3 Cancer Genetics, Genomics and Systems Biology Laboratory, Basic and Translational Cancer Research Center (BTCRC), 1516 Nicosia, Cyprus; a.zaravinos@euc.ac.cy; 4 Department of Life Sciences, School of Sciences, European University Cyprus, 1516 Nicosia, Cyprus

**Keywords:** glioblastoma, YY1, pathogenesis, resistance, immune evasion, targeted therapies

## Abstract

**Simple Summary:**

This report explores the multifaceted role of the transcription factor Yin Yang 1 (YY1), which is overexpressed in many cancers and involved in the regulation of their development. YY1 is expressed in glioblastoma multiforme (GBM) and is involved in its resistance to current therapies. Recent findings provide new insights into the development of targeted therapies designed to inhibit YY1 in tumor growth and resistance to chemotherapy and immunotherapy. Hence, as anti-cancer immunotherapy has gained momentum with the development of new immunotherapeutic strategies approved by the FDA for various cancers, targeting YY1 in combination with these new approved immunotherapies should result in an effective treatment of the GBM.

**Abstract:**

The transcription factor Yin Yang 1 (YY1) plays a pivotal role in the pathogenesis of glioblastoma multiforme (GBM), an aggressive form of brain tumor. This review systematically explores the diverse roles of YY1 overexpression and activities in GBM, including its impact on the tumor microenvironment (TME) and immune evasion mechanisms. Due to the poor response of GBM to current therapies, various findings of YY1-associated pathways in the literature provide valuable insights into novel potential targeted therapeutic strategies. Moreover, YY1 acts as a significant regulator of immune checkpoint molecules and, thus, is a candidate therapeutic target in combination with immune checkpoint inhibitors. Different therapeutic implications targeting YY1 in GBM and its inherent associated challenges encompass the use of nanoparticles, YY1 inhibitors, targeted gene therapy, and exosome-based delivery systems. Despite the inherent complexities of such methods, the successful targeting of YY1 emerges as a promising avenue for reshaping GBM treatment strategies, presenting opportunities for innovative therapeutic approaches and enhanced patient outcomes.

## 1. Introduction

Glioblastoma multiforme (GBM) is an aggressive and malignant form of brain cancer constituting approximately 12–15% of all primary brain tumors. GBM originates from the brain’s glial cells, particularly astrocytes, and ultimately results in cancer cell invasion into the surrounding brain tissue [1]. The pathogenesis of GBM involves a complex interplay of mutations in genes like *TP53* and *EGFR*, leading to uncontrolled cell proliferation, angiogenesis, and its characteristic invasiveness into the surrounding brain tissue [2]. GBM diagnosis often involves a combination of radiographic interpretation of CT or MRI scans, as well as a biopsy to confirm the presence of cancer cells [3]. Current therapeutic modalities for GBM encompass a multimodal approach including surgery, radiation therapy, and chemotherapy with temozolomide (TMZ) [4]. Despite such aggressive interventions, the prognosis of GBM remains poor, with a median survival of approximately 12–15 months following diagnosis, and this underscores the need for novel and effective therapeutic approaches.

**Figure 1:**
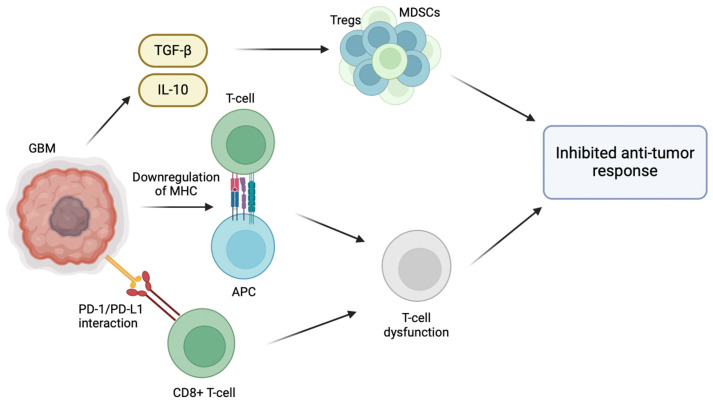
Immunosuppressive tumor microenvironment of glioblastoma multiforme. Various strategies have been employed by GBM cells in the TME to evade the host immune response. GBM cells are able to create an immunosuppressive microenvironment within the tumor through the release of immunosuppressive cytokines such as TGF-β and IL-10, which recruit regulatory T cells (Tregs) and myeloid-derived suppressor cells (MDSCs), inhibiting host anti-tumor immune cell activity. Moreover, effector immune cells are inactivated by the presence of Tregs and MDSCs, allowing GBM cells to proliferate and evade immune surveillance. The interaction between programmed death-ligand 1 (PD-L1) on GBM cells and programmed cell death protein 1 (PD-1) receptors on anti-tumor CD8+ T cells results in T cell inactivation and exhaustion. Lastly, the downregulation of major histocompatibility complex (MHC) molecules on GBM cells compromises the interaction between T cell receptors (TCRs) and antigens presented alongside MHC molecules, inhibiting T cell activation and reducing CD8 T cell immune recognition.

The inherent ability of GBM to evade the host immune response plays a significant role in its pathogenesis, particularly through the modification of its tumor microenvironment (TME) [5]. For instance, GBM promotes an immunosuppressive microenvironment through the release of TGF-β and IL-10, which recruit regulatory T cells (Tregs) and myeloid-derived suppressor cells (MDSCs), inhibiting host anti-tumor immune cell activity [6]. Effector immune cells normally target and destroy cancer cells; however, in the immunosuppressive environment of GBM, effector immune cells are inactivated by the presence of Tregs and MDSCs, allowing immune cells to proliferate and evade immune surveillance [7]. Normally, activated CD8+ T cells are able to effectively identify and target cancer cells. However, the interaction between PD-L1 and PD-1 weakens the T cell response, making them ineffective in combating GBM cells [8]. GBM cells also overexpress immune checkpoint molecules, such as PD-L1, which inhibit T cell activation [7]. The binding of PD-1 receptors on anti-tumor CD8+ T cells to its respective ligands (PD-L1/PD-L2) located on GBM cells results in T cell inactivation and exhaustion [8]. Furthermore, T cell activation depends on the interaction between T cell receptors (TCRs) and antigens presented alongside major histocompatibility complex (MHC) molecules. When this interaction is compromised due to the downregulated MHC molecules on the surface of GBM cells, T cell activation is inhibited and the ability of the immune system to recognize and target them is hindered. Hence, the downregulation of MHC molecules in GBM reduces the CD8 T cell immune recognition of GBM cells and tumor growth [9] (Figure 1).

Yin Yang 1 (YY1) is a ubiquitously expressed transcription factor encoded by the *YY1* gene with the capacity to function both as a transcriptional repressor and an activator [10]. Moreover, YY1 is characterized by its dual nature in cancer, whereby it may be involved in either the promotion or suppression of tumorigenesis [11]. The conserved structure of YY1 is characterized by four zinc finger domains at its C-terminus responsible for transcriptional repression and DNA binding functions of YY1, whereas the N-terminus consists of the YY1 activation domain [10]. YY1 has been reported to be overexpressed in many cancers, contributing to either tumor suppression or progression depending on the cancer type [11,12,13]. As YY1 is overexpressed in GBM, it may play an intrinsic role in the regulation of cell proliferation, viability, migration, metastasis, and resistance to cytotoxic therapies [11,14]. The primary focus of this report is the central role of YY1 expression in GBM in mediating immune evasion and poor prognosis.

## 2. Expression and Activity of YY1 in GBM

Various studies have highlighted the role of YY1 overexpression in the pathogenesis of GBM [15,16]. The research findings outlined below delve into the specific implications of YY1 overexpression in the context of GBM (Table 1).

Chen et al. investigated the role of the long non-coding RNA (lncRNA) small nucleolar RNA host gene 5 (*SNHG5*) in GBM, and demonstrated that it is significantly upregulated [17]. Functional assays have revealed that SNHG5 promotes cell proliferation while inhibiting apoptosis in GBM cells. The investigation further explored the mechanistic regulation of SNHG5, identifying YY1 as a transcriptional activator of SNHG5 in GBM [17]. Notably, the research uncovered that SNHG5 exerts its oncogenic effects in GBM by activating the p38/MAPK signaling pathway [17].

In another study, Liu et al. [18] focused on uncovering the function of the lncRNA prostate cancer-associated ncRNA transcript 6 (PCAT6) in GBM [18]. The findings of the study revealed an increase in PCAT6 expression in GBM tissues and cell lines, attributing its transcriptional activation to YY1 [18]. Subsequent examinations revealed a regulatory sequence involving PCAT6, microRNA-513 (miR-513), and insulin-like growth factor 2 mRNA binding protein 1 (IGF2BP1). PCAT6 was observed to elevate IGF2BP1 expression through miR-513 in a competitive endogenous RNA manner [18]. Both PCAT6 and IGF2BP1 exhibited characteristics of oncogenes, while miR-513 functioned as a tumor suppressor in GBM [18]. The investigation emphasized the pivotal role of the PCAT6/miR-513/IGF2BP1 positive feedback loop in advancing GBM progression by influencing cell proliferation and survival through the AKT signaling pathway. Moreover, the study noted that IGF2BP1 contributes to the stability of PCAT6, establishing a multifaceted regulatory network in the pathogenesis of GBM [18].

Li et al. [19] investigated the expression and functional role of the lncRNA LINC00466 in glioma utilizing reverse transcriptase–polymerase chain reaction (RT-PCR) and observed an increased expression of LINC00466 in glioma tissues and cells, which correlated with reduced overall survival in glioma patients [19]. Moreover, luciferase reporter assays confirmed the direct binding of YY1 transcription factor to the promoter region of LINC00466. Functional assays demonstrated that LINC00466 knockdown suppressed glioma cell proliferation, migration, invasion, and progression of EMT, while promoting apoptosis [19]. Furthermore, LINC00466 was identified as an endogenous sponge for miR-508, negatively regulating its expression. Luciferase and RT-PCR assays revealed checkpoint kinase 1 (CHEK1) as a target of miR-508, and LINC00466 modulated CHEK1 levels by competing for miR-508 [19]. The study suggests that LINC00466 may exert anti-oncogenic effects in glioma through its interaction with the miR-508/CHEK1 axis, presenting LINC00466 as a potential prognostic and therapeutic target for glioma.

Qiu et al. [20] led an integrative analysis of gene expression programs and dependencies in GBM and found that YY1 plays a crucial role as a selective transcriptional dependency in GSCs. YY1 regulates RNA Pol II transcription machinery through interactions with transcriptional CDK9 and the mediation of chromatin loops, acting as a gatekeeper for the transcription cell state [20]. Downstream targets include RNA processing programs involved in RNA splicing and m6A modification. The study proposes targeting the YY1–CDK9 complex or other transcription elongation complexes as a strategy to decrease expression levels of specific genes, inducing interferon responses [20]. Pharmacogenomic modeling suggests compounds targeting transcriptional CDKs as potential inhibitors of the YY1 program, providing a promising approach for treating GBM. The study also highlights the potential of transcriptional CDK inhibitors in reshaping the immunosuppressive microenvironment in GBM, suggesting a combination strategy with immunotherapy for enhanced efficacy in treating patients with GBM [20]. Overall, the findings present a targetable transcription cell state that, when addressed genetically or pharmacogenomically, could be synergistically combined with immunotherapy for GBM treatment.

Gao et al. [21] reported that the A kinase interacting protein 1 (AKIP1) is overexpressed in GBM and correlates with glioma grades. AKIP1 is responsible for interacting with protein kinase A (PKA), an enzyme that plays a key role in cellular signaling pathways promoting tumor progression. Therefore, inhibiting AKIP1 was found to have anti-tumor effects via the inhibition of its interaction with PKA [22]. Furthermore, through its interaction with YY1-mediated Heat Shock Protein 90 Alpha Family Class A Member 1 (HSP90AA1), it stabilizes EGFR. However, HSP90α overexpression reverses EGFR instability caused by AKIP1 depletion, highlighting AKIP1’s significance in GBM progression and revealing a new EGFR regulatory mechanism [22]. In another study investigating the role of guanylate binding protein 1 (GBP1), it was found that EGFR activation induces GBP1 expression in GBM cell lines through a signaling pathway involving Src and p38 mitogen-activated protein kinase [23]. Moreover, YY1 was identified as a downstream transcriptional regulator controlling EGFR-mediated GBP1 expression [23]. GBP1 is important for EGFR-mediated matrix metalloproteinase 1 (MMP1) expression and glioma cell invasion in vitro, establishing it as a potential therapeutic target for inhibiting GBM invasion [23].

You et al. [16] explored the role of the SUMO-specific protease 1 (SENP1)/methyltransferase-like 3 (METTL3)/MYC axis regulated by the transcription factor YY1 in GBM stem cells (GSCs). Research results show that YY1 was highly expressed in GBM tissues and its silencing was subsequently found to reduce the self-renewal ability of GSCs [16]. YY1 promotes SENP1 expression, which in turn promotes the N6-methyladenosine (m6A) modification of MYC mRNA via METTL3, promoting the expression of MYC and enhancing GSC self-renewal [16]. Thus, this study establishes a mechanism whereby YY1 drives GSC tumorigenicity by modulating MYC expression through the coordinated actions of SENP1 and METTL3.

Zhou et al. [24] identified a novel circular RNA, circPTPRF, to be highly expressed in GBM and its role in the promotion of GBM cell proliferation, invasion, and neurosphere formation in both in vitro and in vivo models. Mechanistically, circPTPRF acts as a sponge for miR-1208, relieving its inhibition of YY1 expression and promoting the progressive phenotypes of glioma [24]. This circPTPRF/miR-1208/YY1 axis regulates GBM progression, suggesting a potential role for circPTPRF as a valuable diagnostic and prognostic marker as well as a potential therapeutic target for GBM.

YY1 has been identified to interact with chromatin loops and transcriptional machinery to modulate the expression of genes involved in GBM pathogenesis. Such interactions allow YY1 to directly regulate the expression of genes critical for GBM progression. Qiu et al. identified the role of YY1 in GSCs in regulating RNA polymerase II transcriptional machinery through its interaction with CDK9 and mediating chromatin loops [20]. Genes involved in RNA polymerase (Pol) II-mediated transcription, RNA splicing, and RNA m6A modification were downregulated upon shRNA knockdown and the CRISPR/Cas9 knockout of YY1 in GBM stem cells [20]. Moreover, interactions between YY1 and CDK9 are involved in the regulation of transcription elongation in GSCs, promoting its survival both in vitro and in vivo. Inhibition of the YY1-CDK9 complex led to RNA m6A modification-dependent interferon responses, decreased the infiltration of regulatory T cells, and synergistically increased the response to immune checkpoint therapy [20]. YY1 plays a significant role in the transcriptional regulation of gene expression in GBM and, thus, represents a direct target in GBM treatment strategies.

**Table 1:** Role of YY1 in the pathogenesis of GBM in vitro and in vivo.

Key Findings	Mechanism	Reference(s)
Silencing of YY1 reduced self-renewal ability of GBM stem cells (GSCs)	YY1 promotes SENP1 expression, enhancing m6A modification of MYC mRNA via METTL3, driving GSC self-renewal	You et al. [16]
CircPTPRF/miR-1208/YY1 axis regulates GBM progression	CircPTPRF acts as a sponge for miR-1208, relieving its inhibition on YY1 and promoting GBM cell proliferation, invasion and neurosphere formation both in vitro and in vivo	Zhou et al. [24]
YY1 knockdown in GSCs downregulates genes involved in RNA Pol II transcription, splicing, and m6A modification	YY1 interacts with CDK9 and mediates chromatin loops to regulate transcriptional programs	Qiu et al. [20]
miR-7-5p sensitizes TMZ-resistant GBM cells	Direct targeting of the 3′-UTR of YY1 by miR-7-5p leads to drug sensitization in TMZ-resistant cell lines	Jia et al. [25]
YY1 promotes EMT, invasion, and metastasis of GBM	Regulation of EMT-related genes like E-cadherin, Snail, and Twist1	Bracken et al. [26]
YY1 transcriptionally activates SNHG5 in GBM cells	SNHG5 exerts oncogenic effects by activating the p38/MAPK pathway	Chen et al. [17]
YY1 transcriptionally activates PCAT6, acting as a sponge for miR-513 to upregulate IGF2BP1	PCAT6/miR-513/IGF2BP1 axis promotes GBM progression via AKT signaling	Liu et al. [18]
YY1 directly binds to the promoter of LINC00466 and activates its expression in glioma cells	LINC00466 acts as a sponge for miR-508 to regulate CHEK1 levels	Li et al. [19]
YY1 induces VEGF-mediated angiogenesis	YY1 promotes neovascularization by binding to the VEGF promoter and stimulating angiogenesis to supply oxygen and nutrients to GBM tumor cells	Momeny et al. [27]

## 3. Role of YY1 in the TME of GBM

Recent studies have examined the intricate role of YY1 in modulating the TME, specifically on the extracellular matrix’s remodeling and angiogenesis. Research findings suggest that YY1 exerts an influence over the expression of matrix metalloproteinases (MMPs), enzymes that degrade the extracellular matrix (ECM), facilitating tissue remodeling and creating pathways for tumor cell migration [28]. To infiltrate the intricate ECM of the brain, GBM tumor cells employ MMPs to break down a variety of ECM proteins [29]. Thus, the YY1-mediated activation of MMP expression facilitates the invasion of tumor cells.

YY1 also induces epithelial–mesenchymal transition (EMT), a process vital for metastasis via the suppression of genes like E-cadherin, maintaining the epithelial form, and the activation of genes like *Twist1* and *Snail*, promoting a mesenchymal state [26,28]. E-cadherin, an important cell–cell contact factor, is rarely expressed [30]. The reduced expression of E-cadherin in gliomas may be due, at least in part, to increased YY1 expression and its role in the suppression of E-cadherin. Similarly, Twist1 and Snail, both of which further inhibit E-cadherin expression, are overexpressed in GBM [30].

YY1 promotes tumor angiogenesis by stabilizing the hypoxia-inducible factor-1α (HIF-1α) and activating angiogenic factors like vascular endothelial growth factor (VEGF) and transforming growth factor alpha (TGF-α) [31]. Moreover, YY1 induces vascular endothelial growth factor A (VEGFA) expression by directly binding to the promoter region of VEGF, thereby upregulating their transcription and promoting neovascularization to supply oxygen and nutrients to tumor cells [32]. GBM is a characteristically highly vascularized form of cancer due to the enhanced expression of VEGF [27]. Moreover, studies suggest that the increased expression of VEGF supports malignant progression and is associated with an unfavorable prognosis in GBM [27].

Understanding the direct role of YY1 in these processes holds substantial promise in developing targeted therapies. Such therapies could disrupt the supportive TME, thereby impeding cancer progression and metastasis, potentially offering new avenues for cancer treatment strategies.

## 4. Mechanisms of YY1-Mediated Immune Evasion in GBM

A. Immune Evasion in GBM

GBM tumors employ a variety of mechanisms to evade the immune response, making it difficult to develop and employ effective targeted immunotherapeutic treatments. GBM cells secrete several immunosuppressive factors, such as IL-6, IL-10, TGFβ, and prostaglandin E2 (PGE2), which have been shown to be upregulated in patients exposed to radiotherapy and chemotherapy [7,33]. GBM cells additionally express many cell surface and intracellular inhibitory proteins. For instance, the upregulation of ICAM-1 in GBM and its interaction with lymphocyte function-associated antigen 1 (LFA-1) on myeloid cells promotes the migration of these cells into the tumor, thereby enhancing local immunosuppression. Moreover, the accumulation of MDSCs within GBM tumors contributes to further suppression via the expression of various immunosuppressive molecules, such as TGF-β and arginase, which target and inhibit anti-tumor T cells [34]. GBM cells also increase the expression of non-classical MHC class I molecules, such as HLA-G, to prevent foreign antigen recognition and subsequent T cell-mediated cell destruction [35]. Moreover, the expression of indoleamine 2,3-dioxygenase (IDO) in GBM promotes the differentiation of naïve CD4 T cells into Treg cells, and thus promotes a less effective immune response towards the tumor cells [34]. Lastly, GBM cells upregulate the expression of PD-L1 to further drive immune suppression and promote tumor survival [34]. Due to the multifaceted role of the immune response against GBM, a number of approaches have been proposed to overcome GBM-induced immunosuppression. Such strategies include, but are not limited to, the following: vaccination with various peptides; blockade of inhibitory M2 TAMs by blockade of CSF-1/CSF-1R interaction; immunotoxins targeting TAMs; blockade of TGF-beta; immune checkpoint blockade; engineered CAR-T cells; oncolytic virotherapy and combination of immunotherapy with standard therapy [34]. Combination therapies involving the aforementioned strategies, namely, those that maximize the effective penetration of the blood–brain barrier (BBB), represent a promising approach towards GBM therapy.

In addition to the secretion of immunosuppressive factors and the infiltration of suppressive cells, the hypoxic environment of the TME itself induces the activation of the HIF-1α transcription factor, which plays a crucial role in the adaptation of tumor cells to the low-oxygen environment [36]. The activation of HIF-1α in GBM promotes the differentiation and activation of Tregs, thereby facilitating tumor progression [36]. Furthermore, the activation of HIF-1α leads to the increased synthesis and secretion of VEGF, a potent angiogenic factor. Angiogenesis supplies oxygen and other nutrients to the rapidly proliferating tumor cells while also facilitating the recruitment and infiltration of immunosuppressive cells, such as Tregs and MDSCs [36].

A novel mechanism of immune evasion has been identified in which the serial transplantation of GBM stem cells (GSCs) into immunocompetent hosts led to immune evasion due to epigenetic immunoediting, leading to the establishment of an enhanced immunosuppressive TME [37]. These epigenetic changes promote a myeloid-affiliated transcriptional program, resulting in the increased recruitment of tumor-associated macrophages (TAMs) to the TME. Notably, this transcriptional circuit was also observed in human mesenchymal subtype GSCs, an aggressive subtype of GBM [37]. The authors concluded that reshaping the GBM microenvironment leading to increased recruitment of immunosuppressive TAMs and the establishment of an enhanced immunosuppressive TME represent a novel mechanism by which GBM tumors can evade immune clearance and promote tumor progression [37].

B. YY1-mediated Role in GBM Immune Evasion

Jung et al. [38] emphasize the dual role of the transcription factor YY1 in cancer cells, with its ability to either promote or suppress oncogenic proliferation depending on various factors. YY1 expression is intricately regulated at both the transcriptional and post-transcriptional levels, involving autoregulation, the NF-κB/YY1/microRNA-10a circuit, and the NF-κB/Snail/YY1/RKIP regulatory loop [38,39]. In cancer cells, YY1’s impact extends to the regulation of key anti-apoptotic genes, including Bcl-2, Bcl-xl, Mcl-1, and survivin, known for their involvement in therapeutic resistance and cancer progression. YY1’s inhibition correlates with the downregulation of these anti-apoptotic genes, promoting apoptosis through various pathways, such as the p53 pathway and NF-κB signaling [38]. Additionally, YY1 influences T cell exhaustion, contributing to resistance against cytotoxic T cells [38]. The report explores potential therapeutic strategies targeting YY1, including RNA-based approaches, small molecules, peptides, and specific YY1 inhibitors, highlighting their role as chemo-immuno-sensitizers [38]. The discussion highlights the clinical significance of targeting YY1 and anti-apoptotic proteins in cancer treatment, emphasizing the need for further research to validate clinical applications and identify relevant biomarkers for patient subgroups.

Martinez-Paniagua et al. [40] identified a novel mechanism by which the Bcl-2 inhibitor Obatoclax induces gene regulation in B-cell non-Hodgkin’s lymphoma (B-NHL) tumor cells, leading to the reversal of resistance to TRAIL-induced apoptosis. While Obatoclax was initially designed as a BH3-mimetic, the study suggests additional activities, including the inhibition of the constitutively activated NF-κB survival pathway and the inhibition of various gene products involved in apoptosis [40]. The sensitization to TRAIL apoptosis induced by Obatoclax primarily occurs through the activation of the death receptor DR5, and the study proposes the involvement of both Mcl-1 and the DR5-repressor YY1 in this process [40]. The Obatoclax-mediated inhibition of NF-κB results in the downstream inhibition of several anti-apoptotic gene products, such as Mcl-1, Bcl-XL, cIAP, and XIAP [40]. Interestingly, the Obatoclax-induced inhibition of Bax and Bak, along with the observed dissociation of Bak from Mcl-1, differs from previous reports, suggesting cell-type-specific responses [40]. The proposed dysregulated resistant circuit involving NF-κB, YY1, Mcl-1, and DR5, modified by Obatoclax, offers insights into potential therapeutic strategies for overcoming resistance in GBM cancer cells.

**Figure 2:**
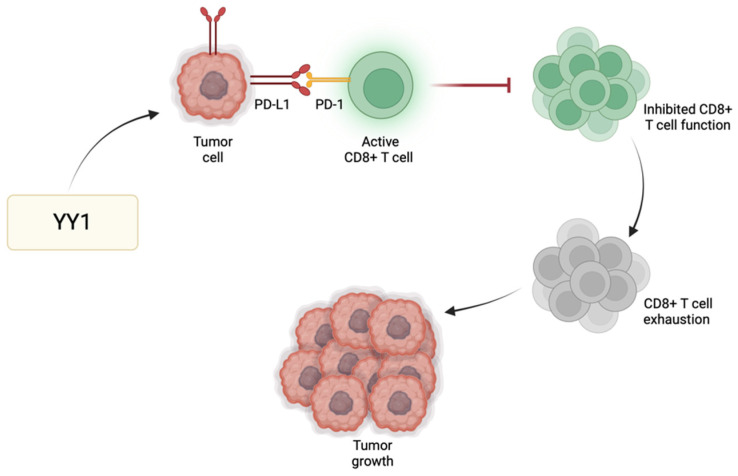
**YY1-mediated immune evasion in GBM.** YY1 has a positive regulatory effect on immune checkpoint pathways, particularly PD-1/PD-L1. As a result of this upregulation, there is heightened interaction between PD-1 and its ligand PD-L1, stimulating the onset and perpetuation of T cell exhaustion. Consequently, T cells lose their effectiveness in initiating an immune response against tumors or pathogens, enabling immune evasion and facilitating tumor growth.

Immune checkpoint regulators, such as programmed cell death protein 1 (PD-1), play a significant role in modulating T cell responses and preventing excessive immune activation. One of the primary immune checkpoint pathways implicated in GBM immune evasion is the interaction between PD-1 and its respective ligands (PD-L1/PD-L2) [41]. PD-L1 expressed by GBM cells binds PD-1 receptors found primarily on activated T cells to suppress T cell activity. GBM cells exhibit heightened levels of PD-L1, leading to effective binding with PD-1 and consequently promoting tumor growth and enabling the tumor cells to resist CD8 cytotoxic cells and evade immune destruction [42]. YY1 has a significant role in the positive regulation of immune checkpoint pathways, particularly those associated with PD-1/PD-L1. For instance, YY1 reduces the expression of miR-34a downstream of p53, resulting in heightened PD-L1 expression via binding to the PD-L1 3′UTR [43]. Additionally, YY1 facilitates PD-L1 expression by reducing PTEN levels through p53 and activating the PI3K/Akt/mTOR pathway [44]. Thus, the role of YY1 in the further enhancement of PD-1 and/or PD-L1 expression may further contribute to the suppression of the anti-tumor immune response, allowing GBM cells to grow and metastasize (Figure 2). The use of immune checkpoint inhibitors to disrupt such inhibitory pathways has been shown to enhance the host immune response against GBM [45]. Since not all tumors respond to immune checkpoint inhibitors, their use alongside drugs targeting YY1 may provide additional synergistic effects to improve the overall therapeutic outcomes for GBM patients.

## 5. YY1-Mediated Regulation of Cytokine Production by GBM

Dysregulation of YY1 activity has been linked to aberrant chemokine expression, disrupting immune cell migration and infiltration to inflammatory sites. For instance, YY1 has been shown to repress the expression of type I cytokines, including interleukin-2 (IL-2) and interferon gamma (IFN-γ) [46]. The combination treatment of GBM cells with IL-2 and recombinant human p53 adenovirus injection (rAd-p53) not only stimulated specific immune responses against GBM cells, but also increased the proliferation of regulatory CD4+ and cytotoxic CD8+ T cells [47]. This combination therapy also enhanced the expression of genes associated with apoptosis, leading to programmed cell death in GBM cells, and leading to tumor regression and prolonged survival in mice [47]. Another research group utilized engineered bone-marrow-derived myeloid cells releasing interleukin-2 (GEMys-IL2) in mice with low-grade gliomas that readily crossed the BBB and entered the TME to stimulate the immune cell response [48]. GEMys-IL2 prolonged survival in mice with low-grade gliomas, suggesting the anti-tumor activity of IL-2 in the innate response within the glioma TME [48]. Moreover, one report established an IFN-γ-related gene signature (IFNGrGS) using genomic data from 429 GBM samples to investigate the role of IFN-γ in GBM [49]. High IFNGrGS scores in samples correlated with increased immune cell infiltration and enhanced innate immune responses, demonstrating the prognostic value and the potential for identifying populations sensitive to immunotherapy and radiotherapy in GBM treatment [49]. Another study demonstrated that the effect of combination treatment with TMZ and IFN-γ on GBM-induced symptoms in rats effectively inhibited tumor growth, improved overall symptoms, modulated oxidative stress, and reduced cytokine levels [50]. Overall, the combined TMZ and IFN-γ therapy proved more effective than monotherapy, suggesting its potential in GBM treatment [50]. Given the diverse role of cytokines such as IL-2 and IFN-γ in the GBM pathogenesis, therapeutics targeting the YY1-mediated downregulation of these cytokines may be beneficial in restoring immune balance and function.

## 6. Therapeutic Implication Targeting YY1 for GBM Immunotherapy

Immunotherapy against GBM has been relatively ineffective, largely due to the highly immunosuppressive TME of GBM tumors. GBM tumors are highly heterogeneous and can be classified by abnormalities in PDGFRA, IDH1, EGFR, and NF1 [51]. Each subtype exerts its own unique immunosuppressive characteristics. The presence and plasticity of GSCs contribute to the immunosuppressive TME through the recruitment of pro-tumorigenic cells [52]. It is noteworthy that GSCs alter their immunomodulatory phenotype according to stemness-regulating stimuli and can mimic the function of other cells such as vascular endothelial cells and immune cells. Hence, these features of GSCs require the further investigation and development of a novel approach to disrupt the bidirectional interactions between GSCs and the TME [52].

Hosea et al. provide a comprehensive overview regarding the potential therapeutic role of targeting YY1 in cancer treatment [28]. Several approaches have been explored to target YY1, including small molecule inhibitors of YY1 protein expression, nucleic acid-based inhibition (miRNAs and lncRNAs), and genome editing techniques like CRISPR/Cas9, among others [28]. Preclinical studies have effectively demonstrated the role of YY1 inhibition in the regulation of tumor growth, apoptosis, and cancer cell sensitization to chemotherapy. Moreover, YY1 used in combination with various immunotherapy approaches may provide an enhanced anti-tumor effect via downregulation of certain immune players, such as PD-L1 [28]. The authors identify the challenges associated with YY1 drug specificity and delivery; however, the use of combination therapies, alternative drug delivery methods, and the use of specific biomarkers may aid in alleviating such challenges.

One challenge in developing YY1-targeted therapies is the specificity of such therapies, as small-molecule drugs targeting YY1 may inadvertently impact other cellular processes, leading to unintended indirect consequences. The high homology between YY1 and YY2, another transcription factor with opposing roles in tumorigenesis, poses difficulties in designing specific drugs [28]. Moreover, while YY1 is generally considered oncogenic, it may exhibit tumor-suppressive functions in certain cancer types, necessitating evaluation in a context-dependent manner [28]. Efficacy concerns include drug resistance, tumor cell heterogeneity, and the tumor microenvironment’s impact. Combination therapies and alternative delivery methods, such as nanoparticles, are now being explored to enhance YY1-targeted therapy outcomes [28]. Additionally, identifying biomarkers predicting treatment response could enable personalized therapeutic plans. Despite these challenges, targeting YY1 remains a promising antitumor strategy, and ongoing research aims to refine drug specificity, enhance efficacy, and improve clinical translation for improved patient outcomes.

Inhibiting YY1 has shown significant promise in hindering tumor phenotypes and reversing resistance [53]. Existing YY1 inhibitors, including siRNA YY1, nitric oxide donors, proteasome inhibitors, and agents targeting survival pathways like NF-kB inhibitors, have demonstrated effectiveness [53]. However, there remains a need for the development of more specific and targeted YY1 inhibitors. Although inhibitors of YY1 have not yet been approved for clinical use in GBM treatment, it is important to note that targeting YY1 could disrupt the signaling pathways that contribute to tumor growth and survival. Moreover, targeting YY1 may prove to be one therapeutic strategy in overcoming resistance to current standard therapies.

Cho and Bonavida [54] discussed the role of EMT in cancer metastasis and growth with a focus on the role of YY1 in regulating EMT. This review explores the indirect regulation of EMT by YY1 through the NF-κB/Snail/YY1/RKIP loop, detailing the mechanisms by which YY1 influences the expression of key genes involved in EMT [54]. Furthermore, the potential direct regulation of EMT gene products by YY1 is discussed, analyzing YY1-binding sites on the promoters of N-cadherin, E-cadherin, vimentin, claudins, and fibronectin [54]. The dysregulated NF-κB/Snail/YY1/RKIP loop in cancer, as well as YY1’s potential as a therapeutic target, is examined, presenting inhibitors such as miRNAs, betulinic acid, and NO donors [54]. The authors conclude by underscoring YY1’s role as an oncogene and its potential as a therapeutic target to inhibit EMT and halt tumor progression.

Qiu et al. [20] investigated the molecular mechanisms underlying resistance to chemotherapy and radiotherapy in GBM by examining gene expression profiles and conducting whole-genome CRISPR/Cas9 screenings in patient-derived GBM stem cells (GSCs), differentiated glioblastoma cells (DGCs), and neural stem cells (NSCs). The researchers identified YY1 as a crucial regulator of GSC stemness, in which YY1 was found to control a specific cellular state characterized by increased RNA polymerase II-mediated transcription and RNA processing [20]. This regulation occurred through YY1’s interaction with transcriptional cyclin-dependent kinases (CDKs) and its role in chromatin loop formation in GSCs [20]. The study demonstrated that YY1 and transcriptional CDKs were essential for GSC survival and the maintenance of stemness both in vitro and in vivo [20]. Furthermore, YY1 knockdown or the simultaneous targeting of transcriptional CDKs triggered interferon responses and enhanced the effectiveness of immune checkpoint therapy, suggesting that YY1-mediated chromatin regulation defines a targeted cell state associated with active transcription and resistance to immunotherapy in GBM [20].

TMZ remains the standard chemotherapy drugs for the treatment of GBM; however, drug resistance proves to be a clinically significant obstacle [55]. Emerging evidence supports the role of microRNAs (miRNAs) in chemotherapeutic resistance as well as in tumorigenesis. One study utilizing RNA sequencing and high-throughput screening demonstrated significant downregulation of miR-7-5p in TMZ-resistant LN229 cells compared to control cells, correlating with GBM recurrence in patients [25]. The overexpression of miR-7-5p sensitized TMZ-resistant cells to the drug and suppressed the stemness of GSCs. Mechanistically, miR-7-5p achieved this by directly targeting the 3′-untranslated region of YY1 [25]. These findings suggest that the inhibition of YY1 via miR-7-5p may be a promising therapeutic approach to prevent resistance to TMZ and enhance long-term drug responses in GBM patients.

Another study employed the use of T7 peptide-decorated exosomes (T7-exo) to target GBM in the brain, enhancing the delivery efficiency of cholesterol-modified siYY1 [15]. In an orthotopic GBM mice model, when combined with TMZ or radiotherapy, the engineered exosomes efficiently delivered cholesterol-modified siYY1, leading to a synergistic inhibition of GBM growth by targeting the knockdown of YY1 [15]. In vitro experiments reveal that T7-siYY1-exo improves sensitivity to chemoradiotherapy and reverses therapeutic resistance [15]. Combining T7-siYY1-exo with TMZ/IR exhibits a synergistic anti-GBM effect, significantly prolonging the survival of GBM-bearing mice. These findings further suggest that T7-siYY1-exo has the potential to address chemoradiotherapy resistance in GBM via the downregulation of YY1.

Gao et al. reported on the role of microRNA-218 (miR-218) in inhibiting the proliferation of human glioma cells via the downregulation of YY1 expression [56]. The authors found that silencing miR-218 in glioma cells led to significant proliferation of glioma cells, whereas the overexpression of miR-218 expression led to the inhibition of glioma cell proliferation. Bioinformatic analysis identified YY1 as a direct target of miR-218; thus, the downregulation of miR-218 promotes the upregulation of YY1 expression and subsequent degradation of p53 [56]. Contrastingly, miR-218 overexpression and subsequent YY1 inhibition led to increased p53 expression and the inhibition of glioma cell proliferation. Study results were then explored further using Western blotting, EdU assays, and CCK-8 assays to validate the findings [56]. The findings demonstrate that miR-218 indirectly regulates p53 tumor suppressor expression through the mediation of YY1, emphasizing the potential therapeutic role of such a pathway in the treatment of glioma.

Neuroblastoma is characterized by aerobic glycolysis, which is thought to support tumor growth and progression. Transcription factors YY1 and MZF1 are thought to play a critical role in the regulation of aerobic glycolysis in neuroblastoma, and thus have been targeted in a novel therapeutic approach [57]. The authors developed a cell-penetrating peptide (MZF1-uPEP) to disrupt the interaction between YY1 and MZF1, which consequently inhibits the transcriptional activation of genes involved in aerobic glycolysis in neuroblastoma cells [57]. MZF1-uPEP administration led to reduced glucose uptake, lactate production, and ATP levels, suggesting reduced glycolysis [57]. Targeting the YY1/MZF1 axis to reduce aerobic glycolysis led to a reduced tumorigenesis and aggressiveness of neuroblastoma, and thus represents a novel therapeutic target.

Despite the promising role of YY1 inhibitors in the treatment of GBM, there are significant challenges that must be considered. Of primary concern is the potential dual nature of YY1 in GBM cancer cell subsets, acting as both a transcriptional repressor and activator. The context-dependent function and effects of YY1 in various cancers complicate the design of inhibitors that can selectively target its oncogenic functions without disrupting its physiological role. YY1 interacts with numerous proteins and regulatory elements, affecting various cellular pathways, making it difficult to develop non-selective and targeted inhibitors that specifically target YY1-mediated pathways in GBM without affecting its essential cellular functions. The genetic diversity of GBM further complicates treatment approaches, requiring a personalized approach to YY1-targeted therapies based on individual tumor profiles. Moreover, drug delivery to the tumor site in the brain is challenging due to the restrictive nature of the BBB. Overcoming this barrier while reducing its impact on healthy tissues remains a significant challenge in the advancement of YY1-targeted therapies. Moreover, the potential to develop resistance to YY1 inhibitors during the treatment process poses a substantial challenge, as tumor cells may adjust and develop strategies to counteract the inhibitory effects of these drugs, diminishing their efficacies. Thus, while YY1 inhibitors hold promise in GBM treatment, addressing challenges related to the multifaceted nature of YY1, molecular complexity, tumor heterogeneity, drug delivery, and potential resistance is paramount for their successful translation into effective therapeutic strategies for patients with GBM.

## 7. Bioinformatics Analysis of YY1 Expression in GBM

Based on the previously discussed data regarding the pleiotropic pro-tumorigenic activities of YY1 on GBM, we examined accessible bioinformatic data sets of cancer tissues from GBM patients to corroborate previous findings. Moreover, we explored the correlative data between high and low YY1 expressions in GBM and the immune cell infiltrates in the TME, as well as the expression of immune checkpoint molecules.

We used RNA-sequencing expression data (level 3) from the TCGA-GBM dataset (https://portal.gdc.com accessed on 6 April 2024), which includes 153 GBM tumors and five normal tissues, to investigate the expression of YY1 in GBM. We also assessed differences in the infiltration of different immune cells between GBM and normal tissue, as well as between YY1-high- (n = 77) and YY1-low (n = 76)-expressing GBMs. In addition, we evaluated the immune scores in these tissues using CIBERSORTx (https://cibersortx.stanford.edu/ accessed on 6 April 2024) [58,59,60,61,62,63,64,65].

**Figure 3:**
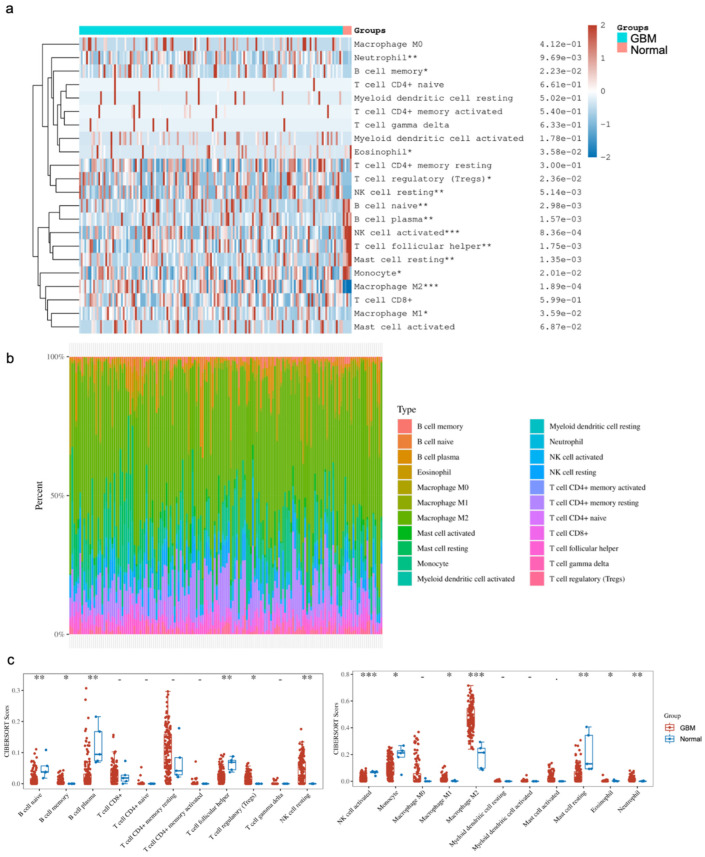
**Distribution of immune cell infiltration (CIBERSORT score) in GBM and normal tissues**. (**a**) Immune cell score heatmap. The different colors represent the expression distribution of CIBERSORT scores between GBM and normal tissue. * *p* < 0.05, ** *p* < 0.01, *** *p* < 0.001. The statistical difference was compared through the Wilcox test. (**b**) The percentage abundance of tumor-infiltrating immune cells in each sample. Different colors represent different types of immune cells. The abscissa represents the GBM samples, and the ordinate represents the percentage of immune cell content in each GBM sample. (**c**) Box plots show the CIBERSORT scores for each immune cell in GBM and normal brain samples. The analyses between normal tissues (n = 5) and GBM tissues (n = 153) demonstrated that there were significant differences in GBM tissues with regard to the frequency of immune cell infiltration. Namely, there were enrichments of the CD4+ T cell memory resting Tregs, NK resting cells, M1/M2 macrophages, eosinophils and neutrophils. In contrast, there were significantly lower levels of naïve B cells, plasma B cells, follicular helper T cells, activated NK cells, monocytes and resting mast cells.

The comparison of immune cell infiltrates between GBM and normal brain tissues focused on subsets of B cells, T cells, NK cells, monocytes and macrophages, myeloid cells, mast cells, eosinophils and neutrophils. Of note, there were subsets that were significantly altered between the two states. Our analysis showed a significant enrichment of CD4+ memory resting T cells, Tregs, NK resting cells, M1/M2 macrophages, eosinophils and neutrophils in GBM, as well as significantly lower levels of naive B cells, plasma B cells, follicular helper T cells, activated NK cells, monocytes and resting mast cells in GBM compared to the normal brain (Figure 3). Provided that the various studies outlined above, as well as in the literature, indicate that YY1 is directly involved in immunosuppression, the bioinformatic findings corroborate the immunosuppressive microenvironment in GBM, and suggest that the overexpression of YY1 plays a role in the regulation of the immunosuppressive nature of the GBM TME.

**Figure 4:**
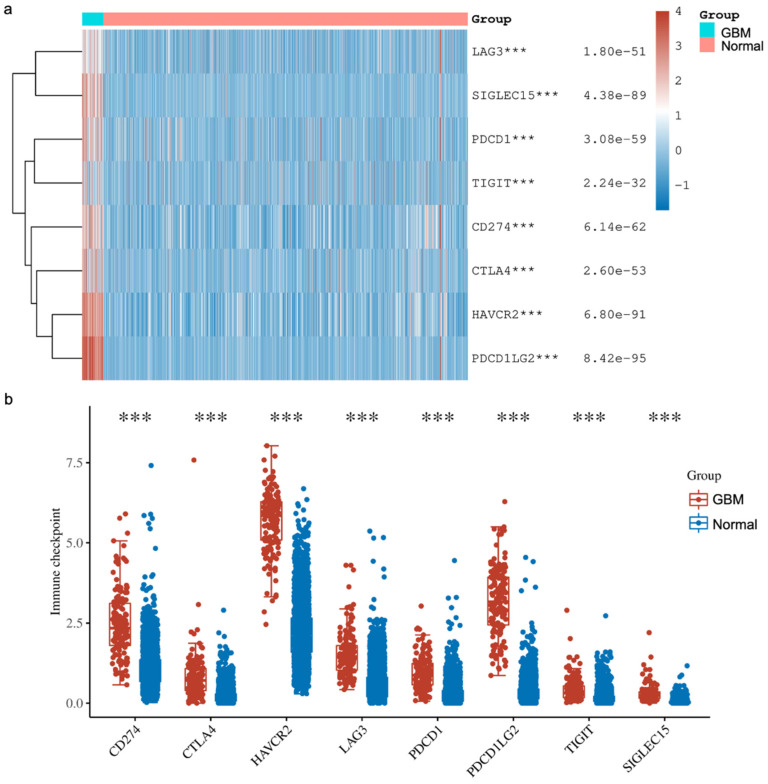
**The expression distribution of immune checkpoints in GBM tissues and normal brain tissues (TCGA, n = 5; GTEx, n = 2642)**. The heatmap (**a**) and scatter plots (**b**) show the expression of 8 immune-checkpoint-related genes in GBM and normal brain samples. The analyses have demonstrated that the expressions of various immune checkpoints are upregulated in GBM tissues compared to the normal tissues. These higher expressions consisted of Sialic acid-binding immunoglobulin-like lectin 15 (SIGLEC15), T cell immunoreceptor with Ig and ITIM domains (TIGIT), Programmed cell death ligand 1 (CD274, PD-L1), Hepatitis A virus receptor 2 (HAVCR2), Programmed cell death 1 (PDCD1, PD1), Cytotoxic T-lymphocyte-associated antigen 4 (CTLA-4), Lymphocyte activation gene-3 (LAG3) and Programmed cell death-ligand 2 (PDCD1LG2, PD-L2). *** *p* < 0.001, asterisks. The statistical difference was compared using the Wilcox test.

In addition, we explored the expressions of various immune checkpoints in GBM and compared them against normal tissues from two databases (TCGA, n = 5 and GTEx, n = 2642). In detail, we downloaded RNA-sequencing expression (level 3) profiles and corresponding clinical information for Sialic acid-binding immunoglobulin-like lectin 15 (SIGLEC15), T cell immunoreceptor with Ig and ITIM domains (TIGIT), Programmed cell death ligand 1 (CD274, PD-L1), Hepatitis A virus receptor 2 (HAVCR2), Programmed cell death 1 (PDCD1, PD1), Cytotoxic T-lymphocyte-associated antigen 4 (CTLA-4), Lymphocyte activation gene-3 (LAG3) and Programmed cell death-ligand 2 (PDCD1LG2, PD-L2) from the TCGA. The analyses were all implemented using *ggplot2* and *pheatmap* in R v4.0.3. Our analysis showed that all the immune checkpoints that we explored were significantly higher in GBM compared to the normal tissue (Figure 4). These findings agree with other reported studies demonstrating that YY1 transcriptionally regulates all of the above immune checkpoints. Briefly, Shao et al. [66] and Chandnani et al. [67] have recently reported the regulation of SIGLEC-15 by YY1. Likewise, Dulal et al. [68] and Ziogas et al. [69] recently reported the regulation of TIGIT by YY1. We and others have reported that YY1 transcriptionally regulates PD-L1 expression [44,70,71]. The expression of HAVCR2 (TIM-3) was reported by Curdy et al. [72]. Furthermore, YY1 has been shown to transcriptionally regulate PD-1 and LAG3 expressions [32,46]. The expression of PD-L2 was reported by Fabrizio et al. [73].

The TME is a complicated system consisting of immune cells, stromal cells, and extracellular factors. Estimating cell components is essential for classifying the distinct tumor immune microenvironment (TIME) phenotype. Furthermore, dissecting the TIME by evaluating cell components plays a significant role in untangling the mechanisms of tumor progression and immune evasion. We further compared the immune infiltration (EPIC scores) between GBM tumors with high levels of YY1 expression (YY1-high GBM) and low levels of YY1 expression (YY1-low GBM). EPIC uses constrained least square regression to estimate six immune cell types, fibroblasts, and endothelial cells. EPIC collects a unique gene expression reference from circulating and tumor-infiltrating cells. Further, it extended its algorithm to evaluate the uncharacterized cancer cells. The score that comes from the EPIC algorithm is an absolute value that can be compared within or across samples.

**Figure 5:**
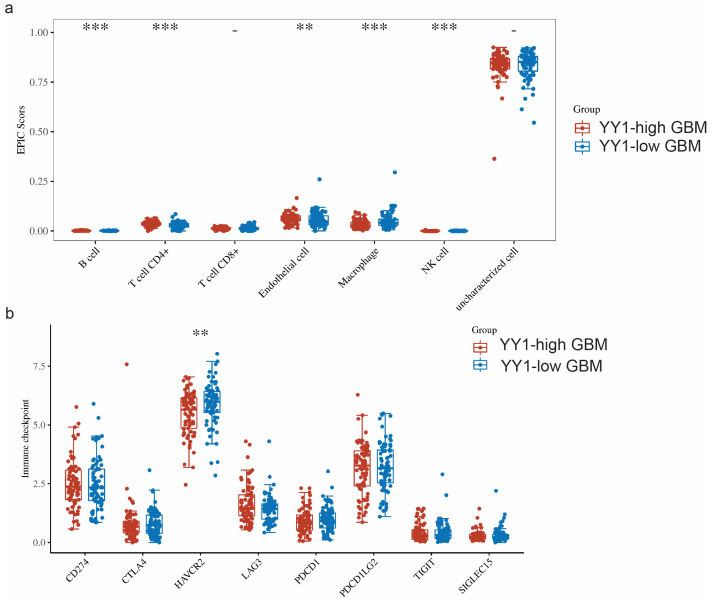
(**a**) Comparison of immune infiltration (EPIC scores) between GBM tumors with high levels of YY1 expression (YY1-high GBM) and low levels of YY1 expression (YY1-low GBM). (**b**) Comparison of the expressions of 8 immune checkpoints (CD274, CTLA4, HAVCR2, LAG3, PDCD1, PDCD1LG2, TIGI, SIGLEC15) in GBM tumors with high and low levels of YY1 expression. ** *p* < 0.01, *** *p* < 0.001. The statistical difference of two groups was compared through the Wilcox test.

We found significant infiltration scores of B cells, CD4+ T cells, endothelial cells and NK cells in YY1-high tumors, as well as a significant infiltration score of macrophages in YY1-low GBM tumors (Figure 5a).

When comparing the expressions of these immune checkpoints between YY1-high- and YY1-low-expressing GBM tumors, we found significantly lower HAVCR2 expression in YY1-high tumors, while no other significant difference could be noted for the rest of the immune checkpoint genes between YY1-high and YY1-low GBMs (Figure 5b). These findings are not easy to explain as the prediction would have been an observed difference between high and low levels of YY1 in the regulation of the checkpoint receptors. However, it is not known at what level the YY1 expression threshold regulates the transcription of immune checkpoints and below which YY1 has no effect.

## 8. Conclusions

**Figure 6:**
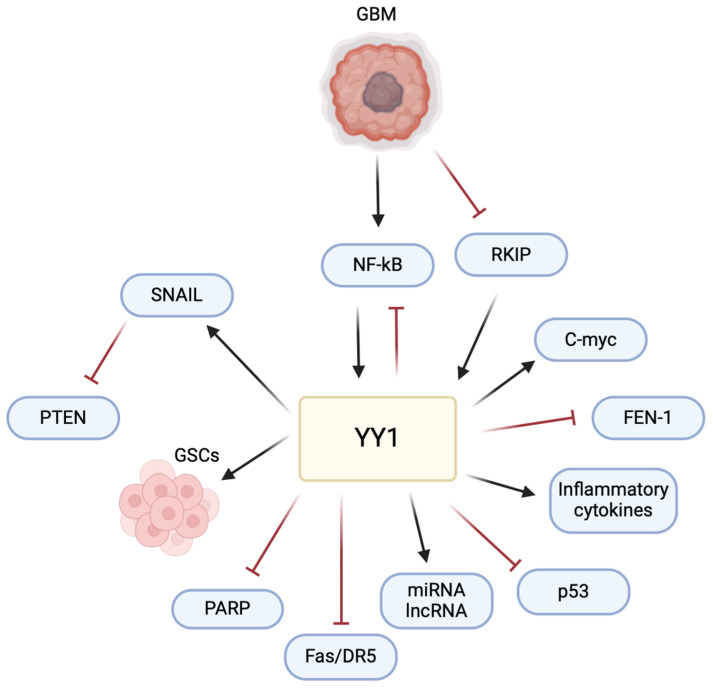
**YY1 plays a central role in regulating multiple pathways involved in GBM pathogenesis.** YY1 activates the expression of SNAIL (repressor of E-cadherin), inducing epithelial–mesenchymal transition (EMT) and promoting invasion and metastasis. YY1 is also critical for the maintenance and self-renewal of glioma stem cells (GSCs), driving tumor initiation, progression, and resistance to therapies. Furthermore, YY1 regulates the expression of various miRNAs, lncRNAs, inflammatory cytokines and the c-Myc proto-oncogene, all of which contribute to cell proliferation and resistance and the pathogenesis of GBM. Conversely, YY1 inhibits pathways involving PARP (poly-ADP ribose polymerase), Fas/DR5 (cytotoxic receptors on CD8 T cells), p53, and FEN-1 (Flap structure specific endonuclease 1), leading to cell proliferation, decreased DNA repair, immune evasion, chemoresistance, genomic instability, and tumor progression. This multifaceted regulation by YY1 highlights its pivotal role in the various pathways that drive GBM pathogenesis.

The multifaceted role of YY1 in the pathogenesis of GBM poses a significant challenge and raises an opportunity for therapeutic intervention (Figure 6). Understanding its intricate mechanisms, particularly in immune evasion and the TME, allows for potential avenues for therapeutic development. The current literature has highlighted the impact of YY1 in GBM, emphasizing its involvement in critical signaling pathways. Studies, such as those examining the interaction of YY1 with AKIP1, SENP1/METTL3/MYC axis, circPTPRF/miR-1208/YY1 axis, and T7-exo-mediated siYY1 delivery, contribute to unraveling YY1’s role in GBM progression. These findings provide insights into potential therapeutic strategies, including targeted drug delivery systems and molecular pathways inhibition, offering potential for more effective GBM treatments in combination with other therapies.

YY1-mediated regulation of PD-L1 in GBM signifies its pivotal role in immune evasion. This understanding presents an opportunity to explore combination therapies, coupling immune checkpoint inhibitors with YY1-targeted drugs to enhance the host immune response against GBM. Additionally, YY1’s influence on cytokine production and the modulation of the TME further emphasizes its impact on GBM pathogenesis.

Collectively, the performed bioinformatic analyses reinforce the role of YY1 in the immunosuppressive TME of GBM. It is noteworthy that these analyses expanded on the pleiotropic role mediated by YY1 in the immunosuppressive TME by regulating various immune cellular compartments through the abundance of immunosuppressive cells and a reduction in anti-tumor immune cells. In addition, the bioinformatic analyses confirmed the overexpression of checkpoint inhibitor receptors on T cells, such as PD-1, TIM-3, LAG-3, TIGIT, and CTLA-4. Furthermore, there is also evidence of the overexpression of inhibitory ligands on tumor cells and other cells in the TME such as PD-L1, PD-L2, and SIGLEC-15. These undoubtedly render the immune effector cells unresponsive to the tumor, which is difficult to overcome using currently approved checkpoint inhibitors. Combination therapies are warranted to overcome the immune evasion of GBM, including targeting YY1. Nevertheless, while the bioinformatic analysis provides correlative evidence between YY1 and the GBM TME, further studies are necessary to establish the direct causal relationship, if any, between YY1 expression and the regulation of immune cell infiltration and checkpoint molecule expression in the GBM TME. Such studies could involve in vitro and in vivo models with YY1 overexpression or knockdown, coupled with immune profiling and functional assays.

Further investigations into the detailed molecular mechanisms underlying the role of YY1 are essential, allowing for personalized medicine strategies that can effectively tackle the diverse nature of GBM and enhance patient outcomes. Translational studies focusing on the practical implementation of YY1-targeted therapies in the clinical setting hold the potential to advance treatment strategies for GBM. Thus, the comprehensive exploration of YY1 in GBM provides a foundation for innovative therapeutic approaches. Despite the complexities and challenges, targeting YY1 holds promise in transforming GBM treatment strategies and improving patient outcomes. As research continues to uncover the nuances of YY1’s involvement in GBM pathogenesis, the prospect of developing successful YY1-targeted therapies becomes more possible.

## Data Availability

All bioinformatic data provided in the text.

## Abbreviations

AKIP1	A kinase interacting protein 1
BBB	Blood-brain barrier
CHEK1	Checkpoint kinase 1
circPTPRF	Circular RNA PTPRF
CRISPR/Cas9	Clustered regularly interspaced palindromic repeats (CRISPR)/Cas9
EMT	Epithelial-mesenchymal transition
GBM	Glioblastoma multiforme
GSCs	Glioblastoma stem cells
HIF-1α	Hypoxia-inducible factor-1α
IGF2BP1	Insulin-like growth factor 2 mRNA binding protein 1
IFN-γ	Interferon gamma
IL-2	Interleukin-2
lncRNA	Long non-coding RNA
MDSCs	Myeloid-derived suppressor cells
METTL3	Methyltransferase-like 3
miR-1208	microRNA 1208
MMPs	Matrix metalloproteinases
MHC	Major histocompatibility complex
NF-κB	Nuclear factor-κB
PD-1	Programmed cell death protein 1
PD-L1	Programmed death-ligand 1
PD-L2	Programmed death-ligand 2
PCAT6	Prostate cancer-associated ncRNA transcript 6
RT-PCR	Reverse transcriptase-polymerase chain reaction
SENP1	Sentrin-specific protease 1
SNHG5	Small nucleolar RNA host gene 5
TCRs	T cell receptors
TGF-α	Transforming growth factor alpha
TMZ	Temozolomide
TME	Tumor microenvironment
Tregs	Regulatory T cells
VEGF	Vascular endothelial growth factor
